# Dispersion Mechanism of Conductive Phase Materials and Micro-Mechanical Properties of ERCC

**DOI:** 10.3390/ma19071411

**Published:** 2026-04-01

**Authors:** Qiaoling Min, Mengxi Zhang, Da Feng, Yinpeng He, Honggang Li, Yixin Wang

**Affiliations:** 1 Hunan Provincial Key Laboratory of Hydropower Development Key Technology, Changsha 410014, China; 2 State Key Laboratory of Hydraulic Engineering Intelligent Construction and Operation, Tianjin University, Tianjin 300350, Chinahyp@tju.edu.cn (Y.H.); yx_w@tju.edu.cn (Y.W.); 3 School of Water Resources and Hydropower Engineering, Xinjiang University of Technology, Hetian 848023, China; 4 School of Civil Engineering and Water Resources, Qinghai University, Xining 810016, China

**Keywords:** cold regions, electrically conductive roller-compacted concrete (ERCC), dispersion mechanism, mechanical properties, mesoscopic simulation

## Abstract

Temperature control and crack prevention are crucial for mass concrete structures in cold regions. Electrically conductive roller-compacted concrete (ERCC) provides a promising route to shift surface temperature regulation from passive protection to active control. To develop an ERCC material suitable for engineering applications, this study first established a quantitative relationship between interparticle interaction energy and particle spacing to elucidate the effect of carbon black (CB) dispersion and agglomeration on concrete performance. The dispersion quality of CB was then evaluated by sedimentation tests, UV absorbance, and resistivity measurements. The absorbance of CB suspensions containing PCE, SDS, and TA increased by 79.9%, 80.1%, and 100.4%, respectively, compared with the suspension without dispersant, and TA gave the lowest mortar resistivity. Mechanical tests and mesoscopic simulations showed that coarse aggregate volume fraction and CB dosage had stronger effects on the compressive strength and elastic modulus of ERCC than aggregate gradation and specimen size. After calibration using the ERCC-2-TA mixture, the average errors between simulation and experiment were 0.7% for compressive strength and 0.4% for elastic modulus. For engineering applications, the recommended ERCC parameters were a coarse aggregate volume fraction of 40%, a CB content of 4–5% and a water-to-binder ratio of 0.45–0.50 for roads, and a CB content of 8% with a water-to-binder ratio of 0.55 for dams.

## 1. Introduction

In recent years, with advances in construction technologies and hydraulic engineering development, dam construction in China has gradually expanded into cold and high-altitude regions. Compared with areas of mild climate, hydraulic engineering projects in cold regions face adverse conditions such as large diurnal temperature variations, extremely low temperatures, and long winter shutdown periods, which pose severe challenges to the construction of roller-compacted concrete (RCC) dams—particularly to thermal insulation and crack prevention for overwintering layers [1,2]. To address these issues, researchers have improved insulation performance from perspectives including the development of insulation materials [3] and the design of insulation schemes [4]. However, these approaches are typically costly and involve relatively complex construction procedures. In essence, they remain “passive protection” measures aimed at reducing surface heat loss. Therefore, it is necessary to develop a functional dam-building material that can satisfy the in-service performance requirements of dam structures while enabling autonomous temperature regulation, achieving a transition of dam surface temperature control from “passive protection” to “active control.” Such a breakthrough is expected to fundamentally resolve temperature-control challenges for high concrete dams in cold regions.

Under the general trend of concrete materials evolving toward multifunctional and intelligent systems [5,6], electrically conductive concrete has emerged as a new type of functional material. It is typically composed of cementitious materials, conductive phases, and aggregates, and integrates electrothermal performance with mechanical properties, showing promising engineering applications and value in snow and ice melting, thermal insulation, and heating [7]. The incorporation of conductive phases leads to pronounced differences between conductive concrete and conventional concrete. Among available conductive phases, carbon black (CB) is low-cost and readily accessible; a clear understanding of how CB affects material performance is therefore important for promoting engineering applications. He et al. [8] incorporated nanocarbon black material into concrete to prepare conductive concrete with self-heating capabilities. They investigated the changes in the mechanical, electrical, and microstructural properties of the concrete under different curing conditions, demonstrating that this type of concrete exhibits significant superiority in resisting extremely cold environments. Li et al. [9] introduced both CB and carbon nanotubes into concrete to develop self-sensing Ultra-High-Performance Concrete (UHPC). They revealed the conductive and piezoresistive sensing performance of the concrete under different admixtures, suggesting that integrating the concrete’s piezoresistive self-sensing capability with resistance sensors will facilitate the application and development of cementitious sensors. Hussain et al. [10] blended CB material into recycled aggregate concrete to fabricate UHPC with a compressive strength reaching up to 140 MPa, which also exhibited stable piezoresistive performance, holding great significance for structural health monitoring in marine and coastal environments. Jahanbakhsh et al. [11] used CB as a modifier for asphalt concrete to enhance its electromagnetic sensitivity, and investigated the effects of CB on heating and healing performance through semicircular bending, indirect tensile, and uniaxial compression tests; the results indicated that CB significantly improved the mechanical properties of asphalt concrete. Monteiro et al. [12] experimentally studied the piezoresistive and sensing performance of CB cement-based composites, providing a low-cost active self-sensing material for monitoring compressive strain in structures such as highway bridges. In hydraulic engineering, Zhang et al. [13] developed electrically conductive roller-compacted concrete (ERCC) suitable for rapid roller-compacted construction, and experimentally evaluated the compactability of fresh ERCC and the electrothermal properties of hardened ERCC. Their work preliminarily demonstrated the effectiveness and engineering feasibility of ERCC for temperature regulation, and further proposed a power supply mode to stabilize concrete surface temperature by considering internal temperature conditions and ambient air temperature. Xu et al. [14] conducted self-sensing performance tests on UHPC incorporating CB at different scales. They found that the smaller the CB particle size, the higher the sensitivity of the electrical properties of concrete to its stress state. Through a multi-scale synergistic enhancement model, they suggested combining nanocarbon black and microcarbon black to promote the formation of a conductive network within the concrete, thereby enhancing the self-sensing capability of the UHPC composite. Li et al. [15] combined experiments with mesoscopic numerical simulations to examine how factors such as the interfacial transition zone and aggregate volume affect resistivity, and proposed a quantitative resistivity prediction model for ERCC, providing an important basis for optimized ERCC design.

For electrically conductive roller-compacted concrete, existing studies have focused mainly on electrical performance and temperature-control effectiveness, whereas the coupling among CB dispersion, mechanical behavior, and engineering-oriented mix design still requires further clarification. Accordingly, the present study makes three main contributions. First, an EDLVO-based comparative analysis is introduced to interpret the agglomeration tendency of CB in alkaline cementitious systems and to explain how excessive agglomeration can impair the rheological state of the paste. Second, the dispersion efficiencies of three representative dispersants are evaluated through sedimentation, UV–Vis absorbance, and resistivity tests, thereby establishing an experimental link between dispersion stability and conductive performance. Third, by combining laboratory mechanical tests with a mesoscopic random aggregate model, the influences of coarse aggregate content, water-to-binder ratio, gradation, and specimen size on ERCC are analyzed to develop a practical mix-selection strategy for road and dam applications in cold-region hydraulic engineering. It should also be noted that the present work focuses on comparative mechanistic interpretation and engineering-oriented parameter selection; freeze–thaw durability, abrasion resistance, and long-term field validation remain topics for subsequent study.

## 2. Analysis of the CB Dispersion Mechanism

### 2.1. Interparticle Interaction Energy in Cementitious Systems

The expanded Derjaguin–Landau–Verwey–Overbeek (EDLVO) theory considers the total interparticle interaction energy $V_{T}$ in cementitious systems as the sum of the van der Waals interaction energy $V_{w}$, the electrostatic interaction energy $V_{E}$ and the polar interaction energy $V_{H}$ [16]. For two particles with radii $R_{1}$ and $R_{2}$, the interaction energy between them can be calculated using Equations (1)–(4).

(1) $$V_{T}=V_{w}+V_{E}+V_{H}$$

(2) $$V_{\varpi }=-\frac{A}{6H}\times \frac{R_{1}R_{2}}{R_{1}+R_{2}}$$

(3) $$V_{E}=\frac{4\pi \varepsilon_{\alpha }R_{1}R_{2}\varphi_{0}^{2}}{R_{1}+R_{2}}\times ln\left(1+e^{-\kappa H}\right)$$

(4) $$V_{H}=2\pi \frac{R_{1}R_{2}}{R_{1}+R_{2}}h_{0}V_{H}^{0}e^{\frac{-H}{h_{0}}}$$

where *H* is the separation distance between interacting particles (m), *A* is the effective Hamaker constant (J), *φ*_0_ denotes the particle surface potential (mV), *κ* is the Debye length (10^6^ m^−1^), *ε_α_* is the absolute permittivity of the dispersing medium, *h*_0_ is the decay length and can be taken as 10 nm for hydrophobic particles, and $V_{H}^{0}$ denotes the energy constant of interfacial polar interactions.

The parameters used in the EDLVO calculation are listed in Table 1. The radius of cement particles was taken as a representative value according to the reported particle-size range of Portland cement and the representative grain size reported for cementitious systems [17,18]. The radius of carbon black was selected based on the typical primary particle size reported for specialty carbon black products. The Hamaker constants were selected as representative values from the literature commonly adopted in dispersion-force calculations for cementitious materials [19,20]. The absolute permittivity of water was determined from the vacuum permittivity and the dielectric constant of water at room temperature [21,22]. It should be noted that these parameters were selected for a highly alkaline aqueous environment intended to approximate fresh cement paste. Therefore, the present EDLVO analysis is mainly used as a comparative and semi-quantitative approach to interpret the agglomeration tendency of carbon black, rather than as an exact full-chemistry description of the evolving hydration system. Within a reasonable parameter range, moderate variations in surface potential, Debye length, and Hamaker constant may change the magnitude of the interaction energy barrier but do not alter the comparative interpretation of dispersion versus agglomeration tendency.

It should be noted that the EDLVO analysis adopted in this study represents an idealized description of the interaction between particles in an alkaline aqueous environment. In real cementitious systems, the pore solution exhibits high ionic strength and continuously evolving ionic composition during hydration [19,23]. In particular, the presence of Ca^2+^, sulfate species, cement particles, and early hydration products may alter the electrical double layer, surface potential, dispersant adsorption behavior, and interparticle forces [23,24,25]. Moreover, the progressive formation of hydration products continuously changes the microstructural environment of the system [23,26]. Therefore, the present EDLVO analysis is mainly used as a comparative and semi-quantitative tool to interpret the relative agglomeration tendency of carbon black at the early stage, rather than as an exact physicochemical description of the full hydration process of cement paste [23]. Accordingly, the calculated interaction energy should be interpreted primarily in terms of relative trends, while the effects of hydration evolution and particle–hydrate interactions should be considered as important limitations of the present model [24,26].

**Figure 1:**
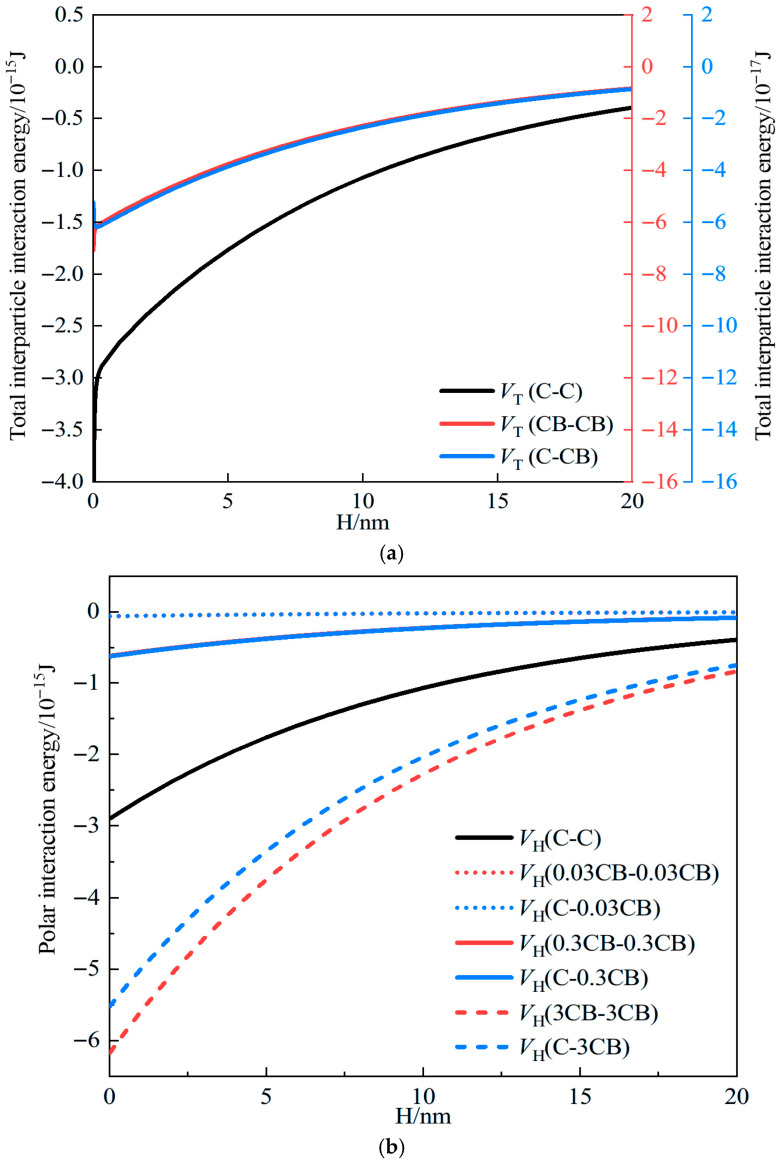
EDLVO theory calculation results. (**a**) Total interparticle interaction energy curves of cementitious systems with fully dispersed CB. (**b**) Effect of CB agglomerate diameter on polar interaction energy under incomplete dispersion.

As shown in Figure 1a, the total interparticle interaction energy indicates that polar interactions dominate the interactions between particles. When CB can be fully dispersed in the ternary system, the attractive interaction between CB particles (VT,CB-CB) is weaker than that between cement particles (VT,C-C), suggesting that CB particles are less prone to poor dispersion caused by excessive attraction. However, complete and uniform dispersion of CB can only be achieved under ideal conditions. During the mixing of cement-based materials in practice, due to the hydrophobic nature of the CB particle surface, CB cannot be fully dispersed and instead forms CB agglomerates with a larger apparent diameter. Here, the agglomerates are approximated as spherical particles, and the dominant polar interaction energy of CB agglomerates with sizes of 0.03 μm, 0.3 μm, and 3 μm in the ternary system is calculated, as shown in Figure 1b. It can be observed that as the agglomerate size increases, meaning more severe CB agglomeration during mixing, the CB–CB attraction becomes stronger. Once it exceeds the cement particle attraction (C–C), the flowability of the composite paste deteriorates, causing the ternary or even multicomponent system to tend toward a coagulated state. Therefore, adopting appropriate measures to modify the hydrophobic characteristics of the CB particle surface, prevent excessive CB agglomeration, and reduce the size of CB agglomerates is an effective approach to improving paste flowability.

### 2.2. Evaluation of CB Dispersion Effectiveness

**Figure 2:**
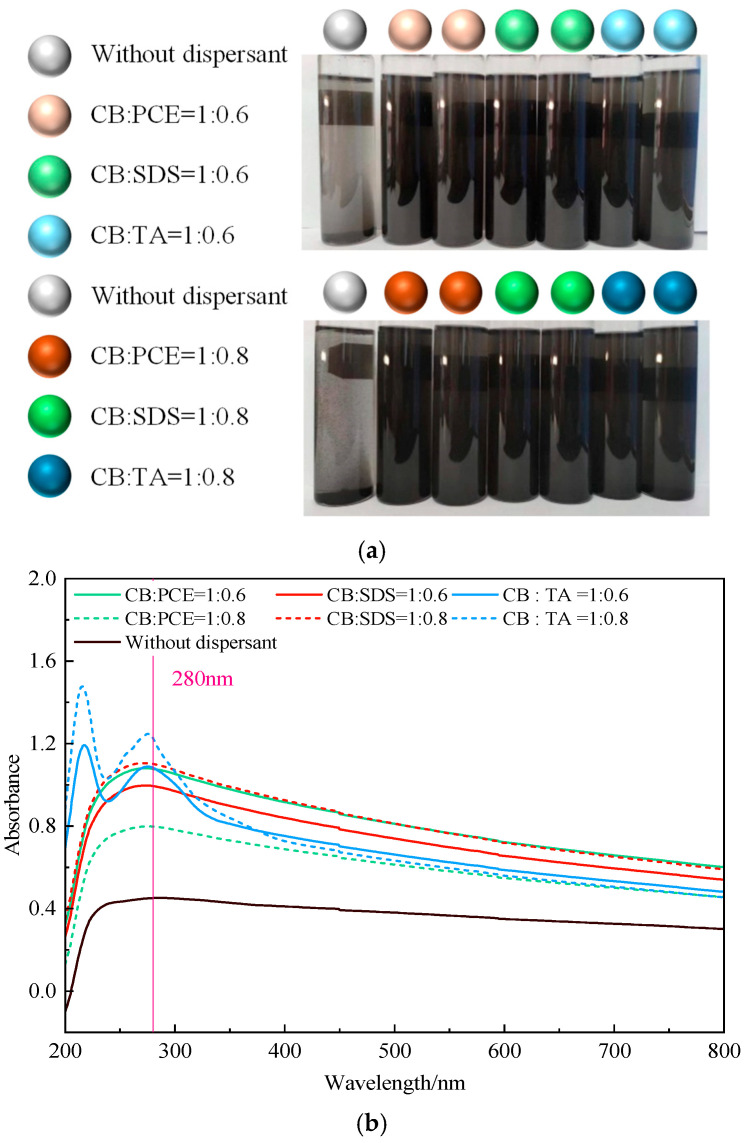

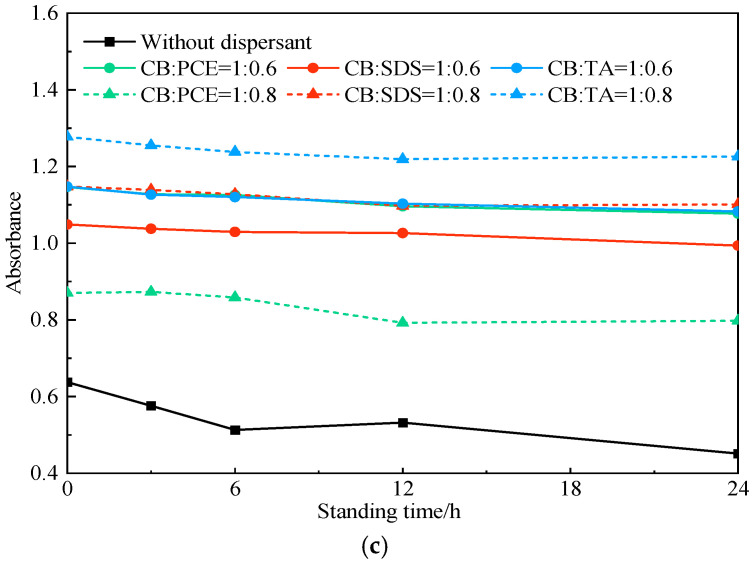
Absorbance of dispersant–CB solutions at different standing times. (**a**) Sedimentation test; (**b**) UV absorbance curves; (**c**) absorbance at 280 nm.

Selecting an appropriate dispersant can effectively reduce the agglomeration of CB particles. To quantitatively evaluate the dispersing effectiveness of sodium dodecyl sulfate (SDS), polyphenolic compounds (TA), and a polycarboxylate superplasticizer (PCE) on CB particles, sedimentation tests were conducted in an alkaline environment using identical 50 mL transparent glass bottles, as shown in Figure 2a. Mixed suspensions of dispersant and CB with different types and proportions were prepared and allowed to stand for 0 h, 3 h, 6 h, 12 h, and 24 h. It was observed that, without dispersant, the CB suspension exhibited non-uniform coloration, and after standing, pronounced particle floating and stratification occurred. In contrast, for CB suspensions containing dispersants, the color distribution was uniform, with no stratification and no visible particulates, indicating that the dispersants promoted CB dispersion and that the dispersant–CB suspensions possessed good stability. To further quantify the dispersion of CB, a TU-1901 double-beam UV–Vis spectrophotometer was used to measure the absorbance of the suspensions at each standing time [27]. The absorbance of dispersant–CB aqueous solutions at different standing times was obtained. Figure 2b shows the effect of wavelength on absorbance, indicating that the absorbance of each group reached a maximum at 280 nm; therefore, a wavelength of 280 nm was adopted in subsequent absorbance tests.

In this study, a carbon black suspension without dispersants was used as the control group to compare the relative effects of different dispersants on the absorbance behavior of the carbon black suspension. It should be pointed out that the control group is used for comparative evaluation, rather than as a strict dispersant matching blank for background subtraction. Therefore, the UV-Vis results in this study are mainly interpreted as a comparative evaluation of the relative dispersibility of different dispersants. As shown in Figure 2c, the absorbance of all turbid suspensions gradually decreased with time. Compared with the case without dispersant, the addition of PCE, SDS, and TA increased the absorbance by 79.9%, 80.1%, and 100.4%, respectively. These results demonstrate that all three dispersants improved the dispersion of CB, among which TA exhibited the best performance and the highest stability.

**Figure 3:**
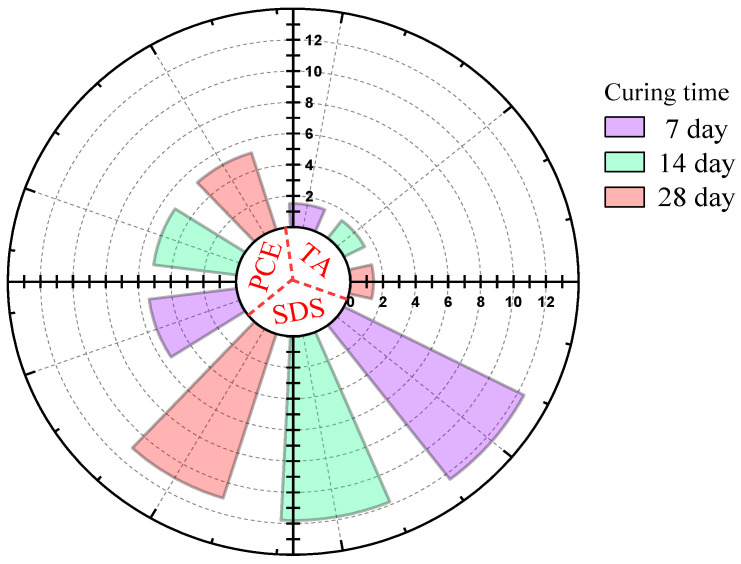
Resistivity of conductive mortar with different dispersants.

For the comparison of electrical resistivity between different dispersants, the mixture was prepared under the same experimental framework, and the key mixture conditions used for comparison remained consistent. Therefore, the observed differences in electrical resistivity may be mainly related to the effect of dispersants on the dispersion of carbon black. Therefore, a 40 mm × 40 mm × 160 mm specimen was prepared. All specimens were stored in a standard curing room at 20 ± 2 °C with a relative humidity exceeding 95% until the respective testing ages (7, 14, and 28 days). The resistivity at curing ages of 7 d, 14 d, and 28 d was measured using the four-electrode method [28], as shown in Figure 3, to further compare the effectiveness of the three dispersants. The results indicate that the resistivity of specimens containing TA is significantly lower than that of the other two dispersants. Accordingly, TA is adopted as the primary dispersant in the subsequent mechanical performance tests. Nevertheless, because the application of SDS is relatively well established, it is also included in this study to provide a meaningful basis for comparative analysis.

## 3. Mechanical Performance Tests and Simulations of ERCC

### 3.1. Mechanical Tests of ERCC

**Figure 4:**
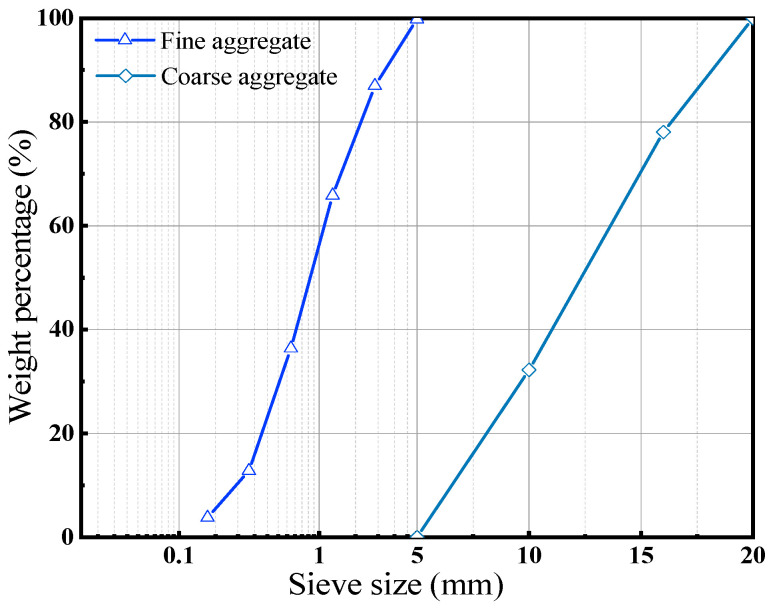
Grading curve of aggregate.

To determine an appropriate mix design for conductive concrete and to clarify how different factors affect its mechanical behavior, a series of laboratory tests were conducted in accordance with the test specifications for hydraulic concrete mix design and practical hydraulic engineering application. Based on the concrete preparation process and the characteristics of conductive concrete, the main raw materials included cement, CB, sand, coarse aggregate, dispersant, and superplasticizer. Ordinary Portland cement PO 42.5 was used. Nanocarbon black with a resistivity of 0.9 Ω·m was adopted as the conductive phase. Natural river sand with a fineness modulus of 3.22 was used as fine aggregate, while the coarse aggregate was first-grade graded aggregate with a particle size distribution of 5–20 mm. Cube specimens with a size of 100 mm × 100 mm × 100 mm were prepared, and three parallel specimens were included for each mix proportion. According to Chinese standard [29], the experimental loading rate was 0.3 MPa/s. The grading curve of the aggregate is shown in Figure 4. The specimens were cured under standard conditions of 20 ± 2 °C and 95% relative humidity for 28 days, and then the compressive strength was measured.

The first part is the experiment on the influence of coarse aggregate volume fraction, aiming to explore the effect of different coarse aggregate volume fractions on the compressive strength of ERCC. The naming convention for samples is “ERCC—Group Number—Dispersant Type—Coarse aggregate volume fraction”; for example, ERCC-1-SDS-10 represents the first group of samples with SDS dispersant and 10% coarse aggregate volume fraction. The second part is an orthogonal experimental design, focusing on the coupling effect of different sand ratios and carbon black content on the mechanical properties of ERCC. The naming convention for samples is “ERCC—Group Number—Dispersant Type—Sand Ratio—Carbon Black Content”. For example, ERCC-1-TA-38-4 represents the first group of samples with TA dispersant, 0.38 sand ratio, and 4% carbon black content. Table 2 summarizes the experimental factors and level used in the orthogonal mixed design.

**Figure 5:**
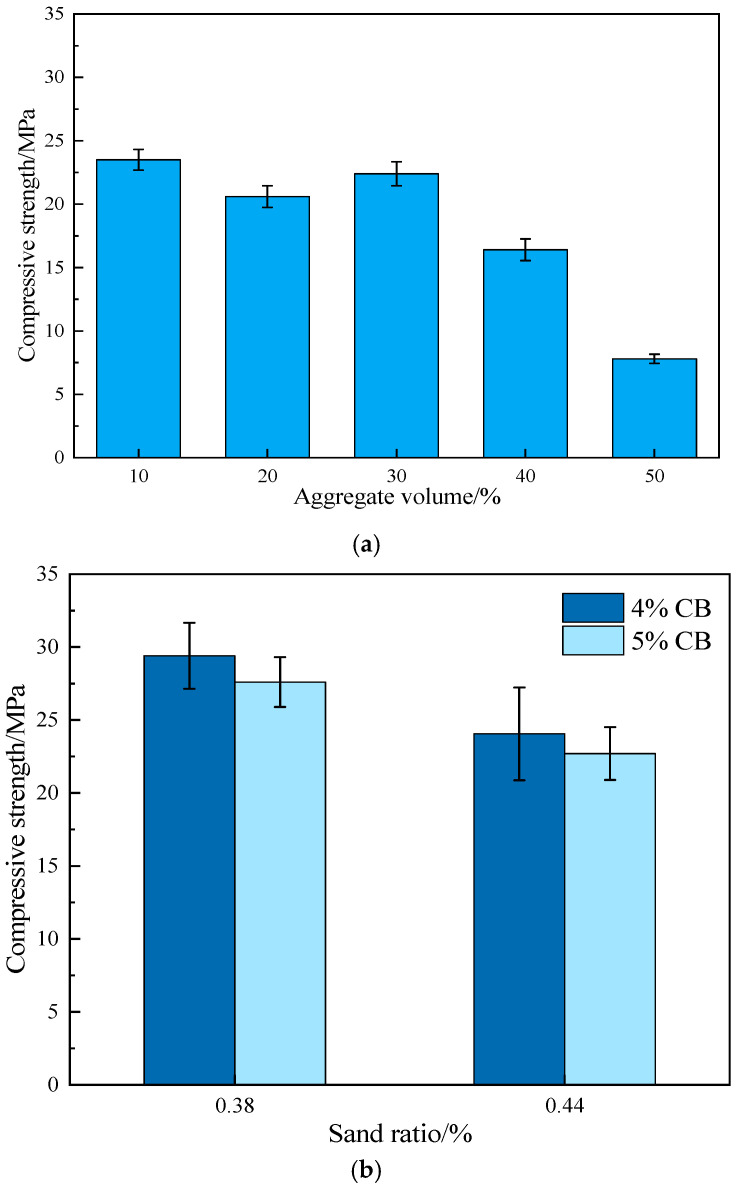
Compressive strength of ERCC under different influencing factors. (**a**) Variation in aggregate volume fraction. (**b**) Variations in sand ratio and CB content.

As shown in Figure 5, the compressive strength results of ERCC indicate that, based on a comparison of test groups 1–5 and under the mix design requirements for RCC dams [30], the compressive strength of ERCC decreases significantly when the aggregate volume fraction exceeds 40%, to the extent that it no longer satisfies the requirements of hydraulic engineering construction. In contrast, the results of the orthogonal tests in groups 6–9 show that an increase in CB content leads to a reduction in ERCC strength. Moreover, when the mortar content is relatively high, the influence of CB content on strength becomes less pronounced. Under these conditions, the compressive strength of ERCC remains generally high and is sufficient to meet engineering requirements.

### 3.2. Mesoscopic Model of ERCC and Its Validation

To further analyze the influence mechanisms of multiple factors on the mechanical properties of ERCC, mesoscopic simulations of mechanical behavior were conducted based on the ERCC mechanical tests. Considering that two-dimensional mesoscopic models offer advantages such as high computational efficiency and fast execution speed, a two-dimensional random aggregate model was established in this study. It is worth noting that the two-dimensional model also has limitations, as it cannot fully reproduce the actual three-dimensional aggregate geometry, spatial interlocking, and ITZ distribution in ERCC. These simplifications may affect the quantitative accuracy of the simulation results, but the model remains suitable for comparative analysis of the investigated parameters. The three-dimensional aggregate gradation curve was transformed into two-dimensional aggregate information using the Walraven–Fuller formula [31], which is expressed as Equation (5):

(5) $$P(D\leq D_{0})=1.065{\left(\frac{D_{0}}{D_{mm}}\right)}^{0.5}-0.053{\left(\frac{D_{0}}{D_{mm}}\right)}^{10}-0.012{\left(\frac{D_{0}}{D_{mm}}\right)}^{60}-0.0045{\left(\frac{D_{0}}{D_{mm}}\right)}^{8.0}+0.0025{\left(\frac{D_{0}}{D_{mm}}\right)}^{100}$$

where *P* is the percentage of aggregates with particle sizes less than or equal to a given size, and *D*_0_ denotes the aggregate particle size. Owing to the inherent simplifications and discrepancies between the two-dimensional random aggregate model and real concrete, circular aggregates were used to approximate actual aggregate shapes in the simulations, and the true aggregate volume fraction was converted into an equivalent area fraction [32]. It was further assumed that aggregate particles do not undergo crushing during loading. Based on these assumptions, the effects of aggregate volume fraction, gradation, and specimen size were systematically analyzed. The elastic modulus of coarse aggregate is 70.0 GPa, and Poisson’s ratio is 0.2. The compressive strength and elastic modulus of the mortar were determined from experiments, while the strength of the interfacial transition zone (ITZ) was obtained through parameter inversion. The ITZ volume fraction was set to 4–7% [33], and the ITZ thickness was taken as 0.5 mm.

**Figure 6:**
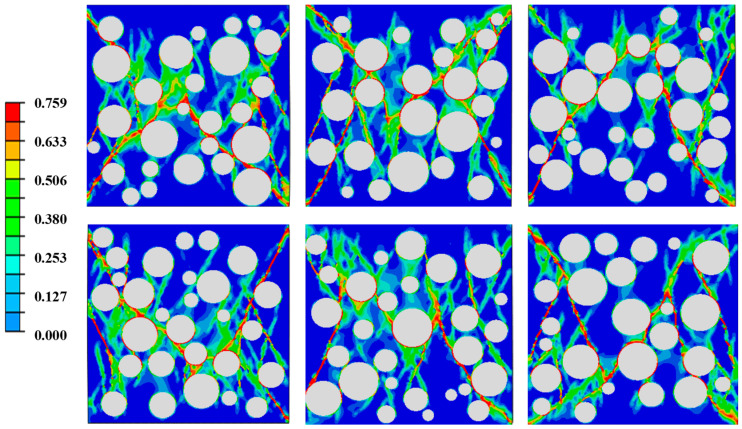
Failure modes of ERCC with different aggregate distributions.

To verify the applicability and accuracy of the two-dimensional random aggregate model, the experimental results of ERCC-2-TA were adopted as the basis for model calibration. According to the inversion results, the strength and elastic modulus of the ITZ were determined to be 0.9 times those of the mortar. Considering the inherent uncertainty of the random aggregate model, six groups of random aggregate models were established. The corresponding failure modes are shown in Figure 6, and the calculated compressive strength and elastic modulus are listed in Table 3. The results indicate good agreement between the experimental and simulation outcomes, demonstrating the validity and feasibility of the proposed model and parameter settings.

It should be noted that the mesoscale model in this study includes several simplifications. First, aggregates were modeled as equivalent circular particles in two dimensions, which simplifies generation and implementation but cannot fully represent the actual 3D geometry, angularity, orientation, and interlocking of coarse aggregates in ERCC. Second, the ITZ was assumed to have constant mechanical properties, ignoring spatial variations caused by local packing, carbon black dispersion, and hydration. Therefore, this mesoscale simulation is mainly a comparative numerical method for analyzing the relative effects of key parameters on the mechanical behavior of ERCC, rather than an exact representation of the real mesostructure. These simplifications may affect quantitative prediction accuracy but do not hinder the model from providing useful mechanistic insights into the experimental trends within the studied parameter range. At the same time, this mesoscale model has been validated primarily using experimental results from a representative ERCC mixture within the range of parameters under investigation. Consequently, the aim of this validation is to demonstrate that, within the modeling framework adopted, the model is capable of reproducing the general mechanical response of ERCC and the trends in its relative damage evolution, rather than to establish a universal calibration and prediction model applicable to all possible mixture ratios.

In this study, mesoscale simulations were established under the assumption of plane stress, with a computational domain size of 100 mm × 100 mm. The numerical analysis adopted the concrete damage plasticity (CDP) constitutive model and simulated it using the nonlinear analysis function in ABAQUS 2022. In the model, the bottom of the model is fixed and a compressive load is applied to the top of the model until the specimen is completely destroyed.

### 3.3. Simulation Analysis of ERCC Mechanical Properties

**Figure 7:**
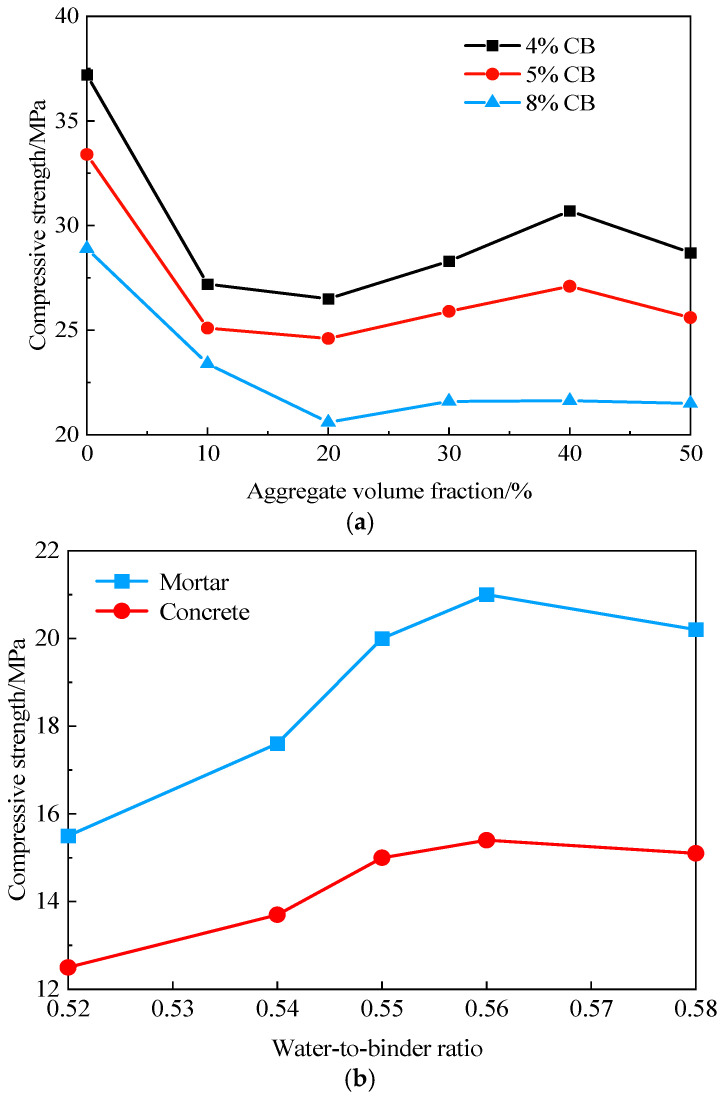
Variation in ERCC compressive strength. (**a**) Variation in aggregate volume fraction. (**b**) Variation in water-to-binder ratio.

As shown in Figure 7a, the influence of coarse aggregate volume fraction on the compressive strength of ERCC is illustrated for a mortar-to-sand ratio of 1:1.5 and CB contents ranging from 4% to 8%. It can be observed that the compressive strength of ERCC first decreases, then increases, and finally decreases again with increasing coarse aggregate volume fraction, with an optimal range of 30–50%. The initial decreasing stage is mainly attributed to the formation of the interfacial transition zone due to the incorporation of aggregates. During compression, the relatively weak ITZ tends to fail first, and as its volume increases with increasing coarse aggregate content, the compressive strength of ERCC decreases. The subsequent increasing stage occurs because the skeletal support effect of coarse aggregates becomes dominant over the influence of the ITZ, so increasing the aggregate volume fraction effectively enhances the strength of ERCC. As shown in Figure 7b, the effect of the water-to-binder ratio on strength indicates that the compressive strength of ERCC first increases and then decreases with increasing water-to-binder ratio. The optimal water-to-binder ratio ranges from 0.55 to 0.58, within which its influence on strength is relatively small.

**Figure 8:**
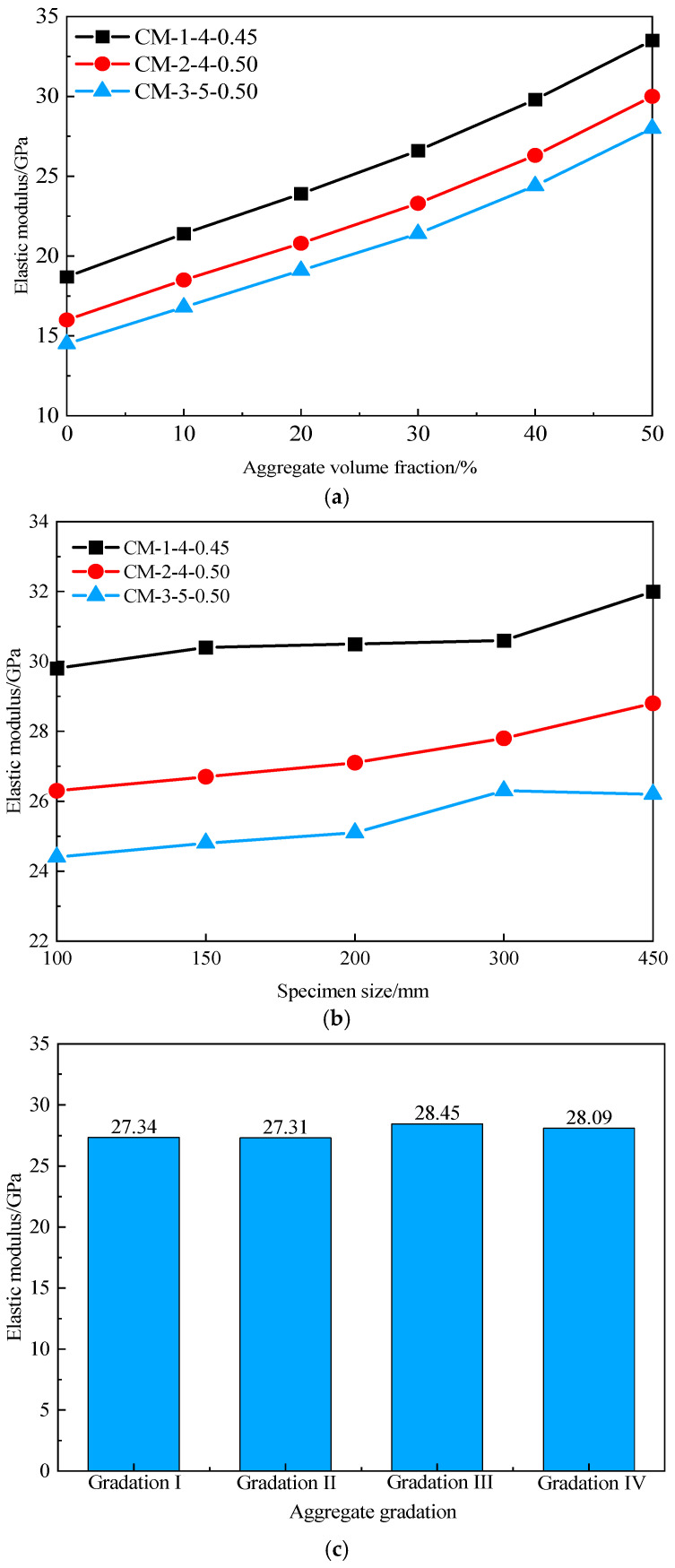
Variation in ERCC elastic modulus. (**a**) Variation in aggregate volume fraction. (**b**) Variation in specimen size. (**c**) Effect of coarse aggregate gradation.

In Figure 8a, the effects of CB content, water-to-binder ratio, and coarse aggregate volume fraction on the elastic modulus of ERCC are presented. Here, CM-1-4-0.45 denotes a mix proportion with a CB content of 4% and a water-to-binder ratio of 0.45. It can be seen that the elastic modulus of ERCC increases with increasing aggregate volume fraction, exhibiting an approximately linear relationship with a consistent slope. This indicates that the influence of coarse aggregates on the elastic modulus of ERCC is independent of the properties of the mortar phase. By comparison, the size effect has a less pronounced influence on the calculated elastic modulus of ERCC. As shown in Figure 8b, the elastic modulus of three groups of ERCC specimens increases by 7.4%, 9.5%, and 7.4%, respectively, with increasing specimen size, which is consistent with the experimental results reported in Ref. [34]. To further analyze the influence of coarse aggregate gradation on the elastic modulus, the aggregate volume fraction was fixed at 40%, and the mechanical properties of ERCC under four different gradations are shown in Figure 8c. The results indicate that aggregate gradation has a relatively small effect on the elastic modulus of ERCC, with a maximum difference of about 4.1%, and its influence is weaker than that of the size effect.

## 4. Engineering Case Analysis

**Figure 9:**
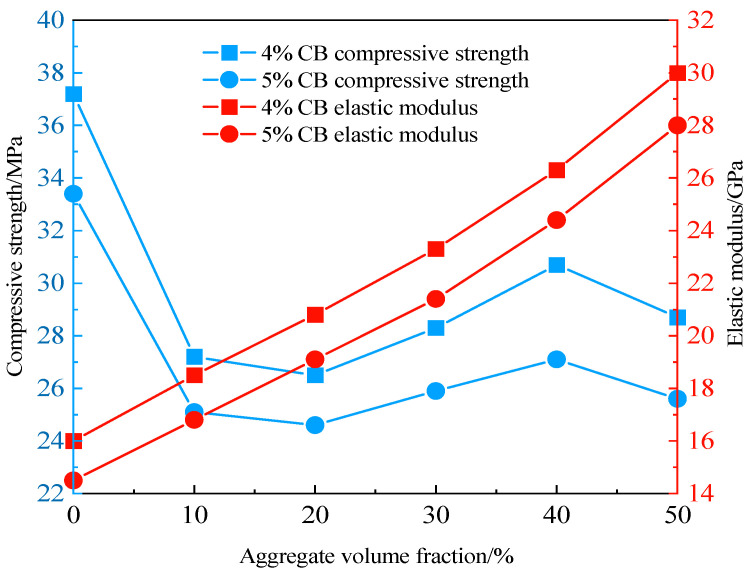
Variations in ERCC compressive strength and elastic modulus with aggregate volume fraction.

A certain water conservancy project is located in a cold region and is greatly affected by the climate of the Qinghai–Tibet Plateau. The maximum temperature difference between day and night can reach 20–25 °C, and the annual average temperature can be as low as 0.7 °C. Extreme weather events such as cold waves are frequent. To verify the applicability and generalizability of ERCC under cold-region climatic conditions, the main access road of this project was selected as a test section for analysis. The design requirements specify that the pavement should reach the C25 concrete strength grade at 28 d, with a pavement thickness no less than 18 cm. The resistivity of the ERCC surface layer should not exceed 30 Ω·m, and the compressive strength of the concrete in the underlying insulation layer at 7 d should be no less than 2.0 MPa. Based on experimental tests and mesoscopic simulations, the effects of multiple factors on the compressive strength and elastic modulus of ERCC were analyzed. The 28 d mechanical test results together with the mesoscale simulation results indicate that CB content and coarse aggregate volume fraction have the most significant influence on the mechanical properties of ERCC. To ensure favorable compressive strength and elastic modulus, it is therefore necessary to first determine an appropriate coarse aggregate volume fraction and a relatively low CB content. As shown in Figure 9, a coarse aggregate volume fraction of 40% can simultaneously ensure a relatively high elastic modulus and compressive strength.

**Figure 10:**
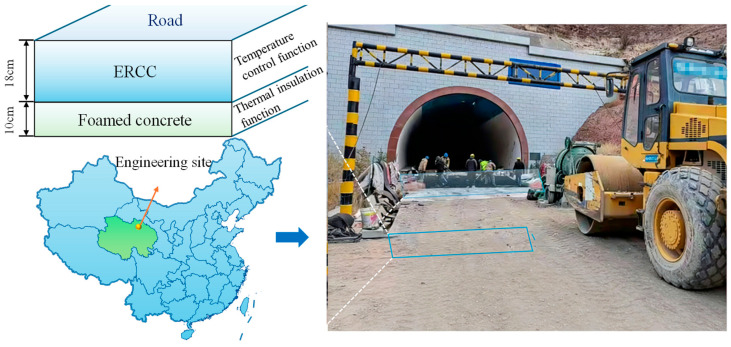
Construction process of ERCC demonstration project in the cold region.

According to relevant codes and engineering application requirements [35,36], the mortar-to-sand ratio is 1:1.5 for road applications and 1:3 for dam construction. For roads, a CB content in the range of 4–5% results in relatively low resistivity while maintaining good compressive strength and elastic modulus, whereas a CB content of 8% is adopted for dam construction. For roads, ERCC prepared with a water-to-binder ratio of 0.45–0.50 exhibits favorable workability and strength, while the water-to-binder ratio needs to be increased to 0.55 for dam construction. When the mortar-to-sand ratio is relatively low, using TA instead of SDS as the dispersant can improve the compressive strength of ERCC to some extent. The optimized mix proportions are listed in Table 4, and the on-site construction process of ERCC in cold-region engineering is shown in Figure 10. In addition to the laboratory results presented in this study, related published field application results, including pavement temperature rise under heating, drainage-ditch de-icing performance, and economic evaluation, provide preliminary support for the practical electro-thermal performance of this material system under cold-region service conditions [37].

## 5. Conclusions

Based on the EDLVO mechanism analysis, dispersion evaluation, mechanical tests, and mesoscopic simulations, the following conclusions can be drawn:

(1) The EDLVO analysis indicates that excessive agglomeration of CB intensifies the attractive interaction among CB particles and is therefore the main reason for the deterioration of the rheological state of CB-containing cementitious systems. As the apparent size of CB agglomerates increases, the CB–CB attraction becomes stronger, which promotes coagulation tendency and adversely affects the flowability of the composite paste.
(2) All three tested dispersants improved the dispersion stability of CB in alkaline suspension. At 280 nm, the absorbance of the suspensions containing PCE, SDS, and TA increased by 79.9%, 80.1%, and 100.4%, respectively, compared with the suspension without dispersant, indicating that TA provided the best dispersion performance. Consistent with the mortar resistivity results, the specimens containing TA showed the lowest resistivity among the tested dispersants. Therefore, TA is recommended as the preferred dispersant for the investigated ERCC system.
(3) The mechanical properties of ERCC are influenced more strongly by coarse aggregate volume fraction and CB dosage than by aggregate gradation and specimen size. The compressive strength first decreases, then increases, and finally decreases again with increasing coarse aggregate volume fraction, and the favorable range is about 30–50%. Among them, 40% is recommended as an engineering-oriented target value because it provides a relatively good balance between compressive strength and elastic modulus.
(4) The mesoscopic model can reasonably reproduce the mechanical response of ERCC within the investigated parameter range. After calibration using the ERCC-2-TA mixture, the average errors between the simulation and experimental results were 0.7% for compressive strength and 0.4% for elastic modulus. This indicates that the model is suitable for comparative analysis of the effects of aggregate distribution and ITZ-related weakening on ERCC mechanical behavior.
(5) For the cold-region engineering scenarios considered in this study, the recommended ERCC mix parameters are as follows: TA as dispersant, coarse aggregate volume fraction of 40%, CB content of 4–5% and water-to-binder ratio of 0.45–0.50 for road applications, and CB content of 8% with a water-to-binder ratio of 0.55 for dam applications. These results provide a basis for the mix design and preliminary engineering application of ERCC in cold-region hydraulic infrastructure.

## Figures and Tables

**Table 1:** EDLVO calculation parameters.

Material Type	Particle Radius/m	Hamaker Constant/J	Particle Surface Potential/mV	Absolute Permittivity /C^2^·J^−1^·m^−1^
Cement particles	20 × 10^−6^	1 × 10^−19^	−0.8	-
CB particles	30 × 10^−9^	6.7 × 10^−20^	−10.4	-
Water	-	4 × 10^−20^	-	6.95 × 10^−10^

**Table 2:** Mix proportions for ERCC mechanical performance tests (kg/m^3^).

Number	Group	Cement	CB	Water	Sand	Stone	Superplasticizer	Dispersant	Curing Age/d
1	ERCC-1-SDS-10	456.0	36.5	250.8	1368.0	270.0	3.7	SDS	28
2	ERCC-2-SDS-20	398.0	31.8	218.9	1194.0	540.0	3.2	SDS	28
3	ERCC-3-SDS-30	340.0	27.2	187.0	1020.0	810.0	2.7	SDS	28
4	ERCC-4-SDS-40	290.0	23.2	160.0	870.0	1040.0	2.3	SDS	28
5	ERCC-5-SDS-50	235.0	18.8	129.0	705.0	1300.0	1.9	SDS	28
6	ERCC-1-TA-38-4	422.2	16.9	190.0	633.3	1034.3	3.4	TA	28
7	ERCC-2-TA-44-4	463.9	18.6	232.0	695.9	886.0	3.7	TA	28
8	ERCC-3-TA-38-5	417.6	20.9	208.8	626.4	1023.0	3.3	TA	28
9	ERCC-4-TA-44-5	467.7	23.4	210.0	701.5	893.3	3.7	TA	28

**Table 3:** Compressive strength and elastic modulus for different aggregate distributions.

Model ID	ERCC-2-1	ERCC-2-2	ERCC-2-3	ERCC-2-4	ERCC-2-5	ERCC-2-6	Average Value	Experimental Value	Error (%)
Compressive strength/MPa	29.4	29.2	28.5	29.5	29.1	30.0	29.3	29.1	0.7
Elastic modulus/GPa	24.2	23.8	23.4	23.9	23.7	24.1	23.9	23.8	0.4

**Table 4:** ERCC mix proportions for cold-region engineering applications.

Application Scenario	CB Content	Water-to-Binder Ratio	Aggregate Volume Fraction	Mortar-to-Sand Ratio	Dispersant
Road	4–5%	0.45–0.5	40%	1:1.5	TA
Dam	8%	0.55	40%	1:3	TA

## Data Availability

The original contributions presented in this study are included in the article. Further inquiries can be directed to the corresponding author.

## Abbreviations

CB	Carbon black
ERCC	Electrically conductive roller-compacted concrete
EDLVO	Expanded Derjaguin–Landau–Verwey–Overbeek
ITZ	Interfacial transition zone
PCE	Polycarboxylate superplasticizer
RCC	Roller-compacted concrete
SDS	Sodium dodecyl sulfate
TA	Polyphenolic compounds
UV–Vis	Ultraviolet–visible spectroscopy
